# Life Expectancy and Mortality After Lower Extremity Amputation: Overview and Analysis of Literature

**DOI:** 10.7759/cureus.38944

**Published:** 2023-05-12

**Authors:** Mohammed Y Qaarie

**Affiliations:** 1 Surgery, Faculty of Medicine, Jazan University, Jazan, SAU

**Keywords:** vascular surgical procedures, comorbidity, mortality, surgical, amputation

## Abstract

Lower limb amputation (LLA) is a major surgical procedure with a significant impact on quality of life and mortality rates as well. Previous studies have shown that mortality rates following LLA can range from 9-17% within 30 days in the UK. This study systematically evaluates and reviews the published literature on life expectancy, mortality, and survival rates following lower extremity amputation (LEA). We have conducted a comprehensive search on Medline, CINAHL, and Cochrane Central databases resulting in 87 full-text articles. After a thorough review, only 45 (52.9%) articles met the minimum inclusion criteria for the study. Our analysis indicated 30-day mortality rates following LEA ranged from 7.1% to 51.4%, with an average mortality rate of 16.45% (SD 14.35) per study. Furthermore, 30-day mortality rates following below-knee amputation (BKA) and above-knee amputation (AKA) were found to be between 6.2% to 51.4%, X= 17.16% ± 19.46 SD and 12.7 to 21.7%, X= 16.15% ± 4.17 SD, respectively. Our review provides a comprehensive insight into the life expectancy, mortality, and survival rates following LEA. These findings highlight the importance of considering various factors, including patient age, presence of comorbidities such as diabetes, heart failure, and renal failure, and lifestyle factors such as smoking, in determining prognosis following LLA. Further research is necessary to determine strategies for improving outcomes and reducing mortality in this patient population.

## Introduction and background

The earliest documented use of lower-limb amputation (LLA) dates back to the Hammurabi era in Babylon, where it was utilized as a form of punishment [1]. Subsequently, the renowned Greek physician, Hippocrates, recommended LLA as a treatment for vascular gangrene [2]. Lower extremity amputation (LEA) has remained a dominant surgical procedure [3]. Despite recent advancements in managing critical ischemia through open revascularization and endovascular techniques, a substantial number of amputations continue to be performed [4].

The primary determinants for the selection of amputation include surgeon preference, physician supply, geographical factors, healthcare delivery systems, and socioeconomic factors [5,6]. The reasons for limb loss can be classified into the following categories: patient-specific issues such as comorbidities, presence of foot lesions, anatomical factors, and healthcare-related factors like access to healthcare and medical insurance [7].

The only absolute indication for this is irreversible ischemia, which can be due to traumatic or non-traumatic etiologies. This ischemia can manifest as gangrene, wet gangrene, spreading cellulitis, arteriovenous fistula, malignancy, severe rest pain with irreversible critical leg ischemia, and paralysis [8]. Factors such as coronary artery disease, nephropathy, and the type of vascular reconstruction surgery have been identified as crucial predictors of the 30-day postoperative mortality rate [9].

Peripheral vascular disease (PVD) and diabetes mellitus (DM) have been identified as primary risk factors for lower limb amputations [10]. Amputations can be classified as cone-bearing (e.g., above-knee and below-knee amputation ) or end-bearing (e.g., grittie stoke, Syme's method) [11]. These procedures are associated with high rates of postoperative mortality (7-23%) and morbidity (15-40%) [12]. Above-knee amputations (AKA) and below-knee amputations (BKA) are commonly performed in patients with failed revascularization, comorbidities, extensive tissue loss, or infection [3]. Following amputation, mortality ranges from 13 to 40% in one year, 35 to 65% in three years, and 39 to 80% in five years [13].

Despite the development and wider availability of novel diagnostic procedures and peripheral vascular interventions, the rates of amputation and subsequent survival have remained relatively unchanged over the past few decades [14]. Ultimately, this highlights the need for continued advancement in this field and the exploration of more efficacious methods to reduce the necessity for amputation and enhance patient outcomes. In our review, we aim to collect almost all published literature and studies disregarding their designs to interpret all the mortality data of patients who underwent amputation in the last hundred years.

## Review

Materials & methods

To achieve a rigorous search for studies relating to Life Expectancy Score in LEA (Lower Extremity Amputation), In February 2021, we searched Medline using OVID, CINAHL, and the Cochrane Central database using a search strategy built on terms that include: "lower limb amputation (LEA), life expectancy, above knee amputation (AKA), below knee amputation (BKA), survival, mortality".

Searching for the used keywords and MeSH terms has been done through Boolean combinations of keywords based on our inclusion and exclusion criteria.

For the identification phase, extensive title and abstract screening were done to detect their relevance. Abstracts that didn't have either mortality or survival rate data of amputees were excluded. Papers that were cited by or used as references in the search set studies were collected and included in our review; after acquiring full-text versions of included studies, deep and extensive reviewing was done. Studies were excluded if they did not present quantitative data on mortality. Moreover, papers were evaluated to determine which outcomes could be meaningfully extracted.

Results

**Figure 1:**
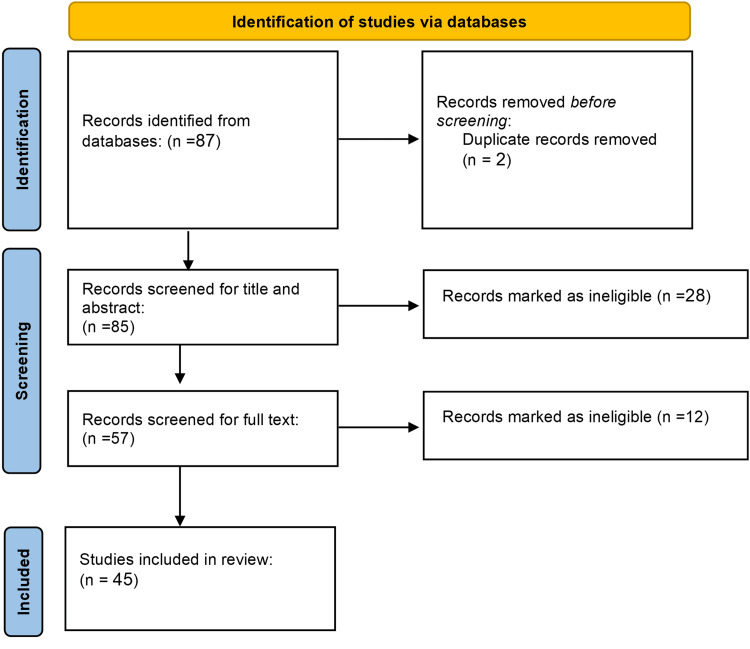
PRISMA flow diagram for included studies

After a comprehensive database search was conducted, PRISMA guidelines for reporting the systematic reviews were followed. Search results led to the identification of 87 full-text articles (as depicted in Figure 1). Upon conducting a thorough review, two articles were found to be duplicates and were subsequently removed, leaving 85 full-text articles. Following the screening process, 28 articles were excluded, while 57 were deemed eligible for further assessment. After a thorough eligibility evaluation, 12 articles were excluded from the systematic review, yielding a final sample size of 45 articles meeting the decided inclusion criteria. The remaining publications (listed in Table 1) represent a total number of 419,149 patients who underwent amputation between the years 1948 to 2010.

30-Day Mortality

Two studies investigated the 30-day mortality rates among individuals with Diabetes 1 and 2 who underwent LEA, reported to be 785 (7.7%) and 729 (7.5%), respectively. The 30-day mortality rates among the 8 studies that did not differentiate the type of amputation ranged from 7.1% to 51.4%, with an average mortality rate of 16.45% (SD 14.35) per study. The studies that investigated the mortality rates following BKA reported a range of 6.2% to 51.4%, with an average mortality rate of 17.16% (SD 19.46). Meanwhile, the studies investigating the mortality rates following AKA reported a range of 12.7% to 21.7%, with an average mortality rate of 16.15% (SD 4.17) per study. One study reported a 12% mortality rate following a combination of BKA and AKA. In the three studies, the mortality rates following toe, Transtibial (TTA), and Transfemoral (TFA) amputations were reported as 1.7%, 7%, and 11.1%, respectively. Four studies reported the mortality rates following trans-metatarsal amputation (TMA) as 1.98%, 2.7%, 4%, and 7%, with an average mortality rate of 3.92% (SD 2.22) per study.

One-year Mortality

The studies that investigated the one-year mortality rates among individuals who underwent LEA with a specified operation reported a range of 9.2% to 64.3%, with an average mortality rate of 33.49% (SD 16.38) per study. Two studies each reported the mortality rates following BKA (range 23-33%) and AKA (range 41-58%) with average mortality rates of 28% (SD 7.07) and 49.5% (SD 12.02), respectively. One study reported a 40% mortality rate following TTA, and another reported a 15.8% mortality rate following both BKA/AKA.

Overall Mortality

Eight studies reported the overall mortality rates among individuals who underwent LEA with a range of 10.6% to 80% and an average mortality rate of 34.24% (SD 26.08) per study.

Discussion

In this systematic review, we have screened the extensive research yield of 85 full-text articles and selected 45 articles that contained representative information about survival and mortality rates in patients who underwent LEA. Very few numbers of published articles explicitly explored the life expectancy of patients after amputation, and it was often only a very small part of every article. Furthermore, only a few studies classify outcomes of patients who underwent amputation depending on the surgical procedures and levels specifically, making it difficult to focus on survival outcomes after either operation. Consequently, we had to include studies where mortality or survival rates are mentioned disregarding than study design.

First, it showed that 30-day mortality has a very wide range of rates with highly variable SD; mortality rates reached up to half of the cohort included. In their retrospective cohort study, the cause of early mortality and low survival rates in the first month and first 1-year after amputation was investigated by Peter Gebuhr et al. [15], where they considered age, pre-existing comorbidities, and re-amputation as causes of early death.

Potential causes of 30-day mortality after amputation include cardiovascular disease, especially in case of declined mobility after AKA, postoperative wound infection, which includes bacterial and fungal infection, and thromboembolic events such as DVT and pulmonary embolism [16-19].

This highlights the importance of considering pre-and postoperative confounding factors that affect short-term survival, which was concluded by P.U. Dijkstra et al., they demonstrated that factors significantly associated with 30-day mortality were age, location admission from the previous peripheral vascular procedure and cerebrovascular disease [20]. It is crucial for healthcare providers to monitor patients following amputation closely and to promptly treat any complications that arise to minimize the risk of 30-day mortality.

Several factors contribute to 1-year mortality after amputation, including medical comorbidities, surgical factors, and rehabilitation outcomes [21]. The authors suggest that this difference may be due to the higher burden of comorbidities in patients undergoing above-knee amputations and the greater rehabilitation challenges associated with this procedure. Previous studies found that amputees who received a prosthesis and participated in a rehabilitation program had lower 1-year mortality than those who did not receive these interventions [22]. Further research is needed to determine the best strategies for improving survival and quality of life in amputees.

Although both BKAs and AKAs result in improved quality of life and survival outcomes compared to limb-saving procedures, it was documented in the literature that there is a difference in postoperative functional outcomes of mobility between the two surgical procedures [23]. Our review also revealed a difference in 1-year mortality between the two major limb amputation types, where AKA patients have a higher mortality rate than BKA patients. This finding goes along with published literature confirming that people who undergo BKA have a higher survival rate than those who undergo AKA [24]. This can be interpreted because BKAs are generally considered less invasive and result in fewer postoperative complications compared to AKAs. On the other hand, BKAs result in improved cardiovascular health; this is believed to be due to the preservation of more of the limb, which allows for a more natural gait and greater mobility, leading to improved physical activity and cardiovascular health [23].

Our review has some limitations. Firstly, the inclusion of studies from different years of publication generalizes our results harder and is not fair because of the variability of healthcare availability, improvement in surgical methodology, and advancement of postoperative follow-up through the years as well as increased incidence of metabolic burden and atherosclerotic changes which not only control the type of operation but also affect post-amputation outcomes.

Secondly, due to the lack of homogeneity of the included cohort, extensive analysis of extracted data had not been done; therefore, we can't conclude solid results of the mortality score of all involved patients.

**Table 1:** Included studies CHF: congestive heart failure, HPT: hyperparathyroidism, IHD: ischemic heart disease, PAD: peripheral artery disease, CAD: coronary artery disease

Study	Cases (n)	Year	Study population	Mean Age (years)	Male Gender	Comorbidity	30 Day mortality	1-Year mortality	Overall-mortality
(Vamos et al., 2010) [25]	84597	1996-2006	England	67.9	0.634	DM	0.076	.	.
(Moxey et al., 2012) [26]	14168	2002-2006	England NHS	70	9336 (65.9%)	.	.	5012 (35.4%)	.
(Jones et al., 2013) [27]	186338	2000-2008	.	77.4±8.2	0.495	Cancer, CHF	0.13	0.481	70.90%
(Wong, 2006) [28]	142	1995-1997	Hong Kong	78.3±7.9	0.42	DM, HPT, IHD	.	.	0.106
(Wiessman et al., 2015) [29]	565	2002-2009	Beersheva, Israel	68.2	0.6	HPT, IHD	37-39.5%	.	.
(Canavan, Unwin, Kelly, & Connolly, 2008) [30]	454	1995-2000	South Tees, England	70	0.663	DM	.	.	.
(Patterson et al., 2012) [31]	306	.	.	74	184 (60%)	.	69 (22.7%)		.
(Beyaz, Güler, & Bağır, 2017) [32]	470	2004-2014	Baskent, Turkey	64.3	299 (63%)	.	16%	36%	0.709
(Scott et al., 2014) [33]	339	2003-2010	Leicester, UK	73 (62-79)	233 (69%)	.	42 (12.4%)	64.30%	.
(Ikonen, Sund, Venermo, & Winell, 2010) [34]	9481	1997-2007	Finland	.	0.7435	DM	.	.	0.897
(Almaraz et al., 2012) [35]	16210	1998-2006	Andalusia, Spain	70.6±11.6	10768 (66%)	DM	.	.	.
(Icks et al., 2011) [36]	444	2005-2009	German	69.1	0.718	DM	245 (55%)	.	.
(Eskelinen, Eskelinen, Albäck, & Lepäntalo, 2006) [37]	1094	1990-2002	Helsinki, Finland	74.2	0.47	DM	.	.	.
(Kolossváry et al., 2015) [38]	32084	2004-2012	Hungary	63.9±11.5	0.65	DM	.	.	.
(J M P de Godoy, de Godoy, Batigalia, Trávolo, & Monteiro, 2005) [39]	50	1993-1998	Sao Jose do Rio, Brazil	67.3	28 (56%)	.	.	22 (44%)	36 (72%)
(Lombardo, Maggini, De Bellis, Seghieri, & Anichini, 2014) [40]	11639	2001-2010	Italy	72.2±14.5	.	DM	.	.	.
(Pande, Kamal, Zaw, & Tin, 2019) [41]	70	2008-2016	Singapore	62.7 (35-88)	45 (64%)	DM, HTN, IHD	.	15.87%	0.486
(Robinson, 1976) [42]	148	1967-1975	Queen Mary Hospital, UK	.	70.25	.	.		.
(Shah et al., 2013) [43]	391	.	.	67.3	0.63	DM, HPT	.	36 (9.2%)	.
(Cruz, Eidt, Capps, Kirtley, & Moursi, 2003) [44]	229	1994-2001	Arkansas, USA	68.6±0.6	221 (96%)	.	12% BKA & AKA		.
(Remes et al., 2008) [45]	210	1998-2002	Turku, Finland	76.3	95 (45.2%)	DM	.	109 (52%)	168 (80%)
(Carmona et al., 2005) [46]	209	1990-1999	Geneva, Switzerland	78±7.5	116 (55.5%)	DM	.	0.324	.
(Mandrup-Poulsen & Jensen, 1982) [47]	310	1971-1979	Denmark	70	181 (58%)	.	55 (18%)		.
(Rosen, Gigi, Haim, Salai, & Chechik, 2014) [48]	188	2007-2010	Tel Aviv, Israel	72	.	.	21.7% AKA, 14.7% BKA	.	.
(Hermodsson, Ekdahl, & Persson, 1998) [49]	112	.	Sweden	.	57 (50.8%)	.	.	.	.
(Lim et al., 2006) [50]	87	2000-2002	Perth, Australia	70.1±14.3	.	DM, HPT, IHD	0.101	0.431	.
(Johannesson et al., 2010)	217	1997-2006	Skane, Sweden	77	112 (52%)	DM	.	86 (40%)	.
(Feinglass et al., 2001	4061	1991-1995	USA	68	.	DM	13.2% AKA, 6.2% BKA	.	.
(Karam, Shepard, & Rubinfeld, 2013) [51]	6839	2005-2008	Detroit, Michigan	.	0.62	HPT,PAD,DM	(6.5%) BKA, (12.7%) AKA	.	.
(Nelson et al., 2012) [52]	9368	2005-2010	USA	67.8±13.5	.	DM	12.8%AKA, 26.6%BKA		.
(Bates et al., 2012) [53]	4153	2003-2004	Mid-West, USA	66.5±11.2	4115 (99%)	.	.	720 (17.34%)	.
(Ciufo, Thirukumaran, Marchese, & Oh, 2019) [54]	4631	2012-2014	USA	64.5	3070 (66.3%)	.	238 (51.4%)		.
(Sandnes, Sobel, & Flum, 2004) [55]	13807	2004-2014	Baskent, Turkey	67±15	0.585	DM	0.492		.
(Mayfield et al., 2001) [56]	5180	1992	USA	65.6	0.99	DM, Renal, CVS, CHD	.	.	0.555
(Kulkarni, Pande, & Morris, 2006) [57]	201	1994-1995	Manchester, UK	69	134 (67%)	PAD/DM	.		.
(Pohjolainen & Alaranta, 1998) [58]	705	1984-1985	Helsinki, Finland	.	323 (46%)	Vascular disease, DM		0.43	.
(Thomas, Perkins, Magee, & Galland, 2001) [59]	41	1991-1998	Reading, United Kingdom	71 (40-91)	37 (90%)	DM, IHD	7 (17%)		.
(Mwipatayi et al., 2005) [60]	43	1999-2002	Capetown, South Africa	59.5 (28-72)	23 (53%)	.	.	.	0.18
(Pollard, Hamilton, Rush, & Ford, 2006) [61]	91	1993-2004	Oakland, USA	64.3 (39-86)	78 (77%)	DM, CAD	0.0198	.	.
(Krause, deVries, Meakin, Kalla, & Younger, 2009) [62]	60	1999-2002	Tennessee, USA	57.9±15	39 (65%)	DM	17 (28%)	.	.
(O’Brien, Cox, & Shortell, 2013) [63]	8878	2005-2010	USA	.	.	.	626 (7.1%)	.	.
(Terashi, Kitano, Tsuji, Hashikawa, & Tahara, 2011) [64]	11	.	.	71 (56-88)	.	DM, PAD	.	.	3 (27%)
Ronald B. Kihn et. al. (1992) [65]	427	1967	Rocky Mountains, USA	.	.	DM	47 (11%)	.	.
Tang et. al. (2009) [66]	84597	.	.	.	.	DM	0.75	0.235	.
Modan et. al. (1998) [67]	101	1948-1974	Israel	58.6±5.4	.	HPT, DM, IHD, CVS	.	.	0.214

## Conclusions

Our review summarizes the last hundred-year literature involving mortality data for almost half a million amputees in approximately 17 countries worldwide. This gives a comprehensive overview of the life expectancy of amputation survivors and encourages researchers, internal physicians, and surgeons to collaborate to prevent amputation at first and eventually improve pre and postoperative comorbidities of amputees.
